# Acellular ex vivo lung perfusate silences pro-inflammatory signaling in human lung endothelial and epithelial cells

**DOI:** 10.1186/s12967-023-04601-w

**Published:** 2023-10-17

**Authors:** Jamie E. Jeon, Lei Huang, Zhiyuan Zhu, Aaron Wong, Shaf Keshavjee, Mingyao Liu

**Affiliations:** 1 Latner Thoracic Surgery Research Laboratories, Toronto General Hospital Research Institute, University Health Network, 101 College Street, PMCRT2-814, Toronto, ON M5G 1L7 Canada; 2 Department of Physiology, Temerty Faculty of Medicine, University of Toronto, Toronto, ON Canada; 3 Department of Thoracic Surgery, Union Hospital, Tongji Medical College, Huazhong University of Science and Technology, Wuhan, 430022 China; 4 Department of Otolaryngology, Affiliated Hospital of Nanjing University of Chinese Medicine, Nanjing, 210000 China; 5 Institute of Medical Science, Temerty Faculty of Medicine, University of Toronto, Toronto, ON Canada; 6 Department of Surgery, Temerty Faculty of Medicine, University of Toronto, Toronto, ON Canada

**Keywords:** Ex vivo organ perfusion, Ischemia reperfusion, Lung transplantation, Cell culture model, RNA sequencing

## Abstract

**Background:**

Ischemia–reperfusion injury is a key complication following lung transplantation. The clinical application of ex vivo lung perfusion (EVLP) to assess donor lung function has significantly increased the utilization of “marginal” donor lungs with good clinical outcomes. The potential of EVLP on improving organ quality and ameliorating ischemia–reperfusion injury has been suggested.

**Methods:**

To determine the effects of ischemia–reperfusion and EVLP on gene expression in human pulmonary microvascular endothelial cells and epithelial cells, cell culture models were used to simulate cold ischemia (4 °C for 18 h) followed by either warm reperfusion (DMEM + 10% FBS) or EVLP (acellular Steen solution) at 37 °C for 4 h. RNA samples were extracted for bulk RNA sequencing, and data were analyzed for significant differentially expressed genes and pathways.

**Results:**

Endothelial and epithelial cells showed significant changes in gene expressions after ischemia–reperfusion or EVLP. Ischemia–reperfusion models of both cell types showed upregulated pro-inflammatory and downregulated cell metabolism pathways. EVLP models, on the other hand, exhibited downregulation of cell metabolism, without any inflammatory signals.

**Conclusion:**

The commonly used acellular EVLP perfusate, Steen solution, silenced the activation of pro-inflammatory signaling in both human lung endothelial and epithelial cells, potentially through the lack of serum components. This finding could establish the basic groundwork of studying the benefits of EVLP perfusate as seen from current clinical practice.

**Supplementary Information:**

The online version contains supplementary material available at 10.1186/s12967-023-04601-w.

## Introduction

Lung transplantation (LTx) is an effective treatment for patients with end-stage lung diseases. However, there are ongoing major issues associated with LTx, such as donor lung shortage and primary graft dysfunction (PGD) after transplantation. Ischemia–reperfusion (IR) is an inevitable process in organ transplantations where a donor organ undergoes static cold preservation followed by warm reperfusion with recipient’s blood at body temperature. Ironically, IR can induce significant injury to the reperfused organ, which can lead to the development of PGD, the major culprit of early morbidity and mortality in LTx recipients [1]. The fear of IR injury and PGD has further limited the use of “marginal” donor lungs, that is lungs that may be transplantable but do not meet the criteria for ideal donor lungs [2]. The imbalance between a high demand and low utilization of donor lungs has created a waitlist mortality of 10–13% [3, 4].

Fortunately, an innovative technology was introduced to address the donor lung shortage: ex vivo lung perfusion (EVLP). It is a platform designed to evaluate marginal lung grafts to increase their utilization. In the Toronto Lung Transplant Program, over 900 marginal donor lung grafts have been assessed with EVLP, of which about 70% of them have been transplanted and yielded good clinical outcomes [5–8]. It has been speculated that the current procedure of EVLP may have some therapeutic effects on donor lung injury [9]. However, convincing evidence and possible mechanisms are largely lacking.

Omics and big data studies have been increasing in the field of organ transplantation, including LTx [10]. One of the categories of omics is transcriptomics, which is an investigation of all RNA transcripts in a given sample [11]. When combined with differentially expressed gene (DEG) analysis and pathway enrichment analysis, transcriptomics data have yielded significant insights into IR and EVLP. For instance, Wong et al. performed a microarray analysis to compare gene expression profiles of human lung biopsies collected pre-/post-LTx and pre-/post-EVLP and identified commonly enriched pathways related to inflammation and cell death after LTx (i.e., IR) and EVLP [12]. Baciu et al. used a multi-omics approach and reported that nutrient and oxidative stress related to inflammation were key responses after LTx. Furthermore, they found increased levels of uric acid and decreased inosine were significantly correlated with worse clinical variables post-LTx [13]. However, the cell types that are responsible for these responses are unknown.

Over the past two decades, cell culture models have been developed to simulate the static cold ischemic storage and warm reperfusion, and they have been used to investigate molecular mechanisms of lung IR injury [14–16] and to test therapeutic interventions [17–21]. Some novel potential therapeutics examined with cell culture models have further been validated with animal studies [18, 21–23]. Saren et al. showed distinct gene expression profiles between human lung endothelial and epithelial cells, which were altered during simulated IR in a cell-type-specific manner, especially after prolonged cold ischemia of 18 h [16]. Recently, these cell culture models were modified to simulate EVLP, and it has been found that adding L-alanyl-L-glutamine to the commonly used EVLP perfusate, Steen solution, can improve basic cellular function and protect porcine lungs during EVLP to yield stable lung function for a prolonged time [24].

Implementing cell culture models provide a great opportunity to determine the mechanisms of IR and/or EVLP in human lung cells. We hypothesize that bulk RNA sequencing can reveal cell-type-specific cellular and molecular responses to IR and/or EVLP using human lung endothelial and epithelial cell culture models.

## Materials and methods

### Cell lines and reagents

Human lung epithelial cells (BEAS-2B) were purchased from ATCC (Manassas, VA), and human pulmonary microvascular endothelial cells (HPMEC) were a gift from Kirkpatrick’s research lab [25]. Cells were passaged in low glucose Dulbecco’s modified Eagle’s medium (DMEM) with 10% fetal bovine serum (FBS) and Pen/Strep (100 U/100 μg/ml) (Thermo Fisher Scientific; Burlington, Canada), and the passages were counted after thawing individual aliquots from the liquid nitrogen storage. For HPMEC, the flasks and plates were coated with 0.2% gelatin at least 2 h before seeding cells according to previously established protocol [24, 25].

### Cell culture models simulating ischemia-reperfusion and ex vivo lung perfusion

**Fig. 1:**
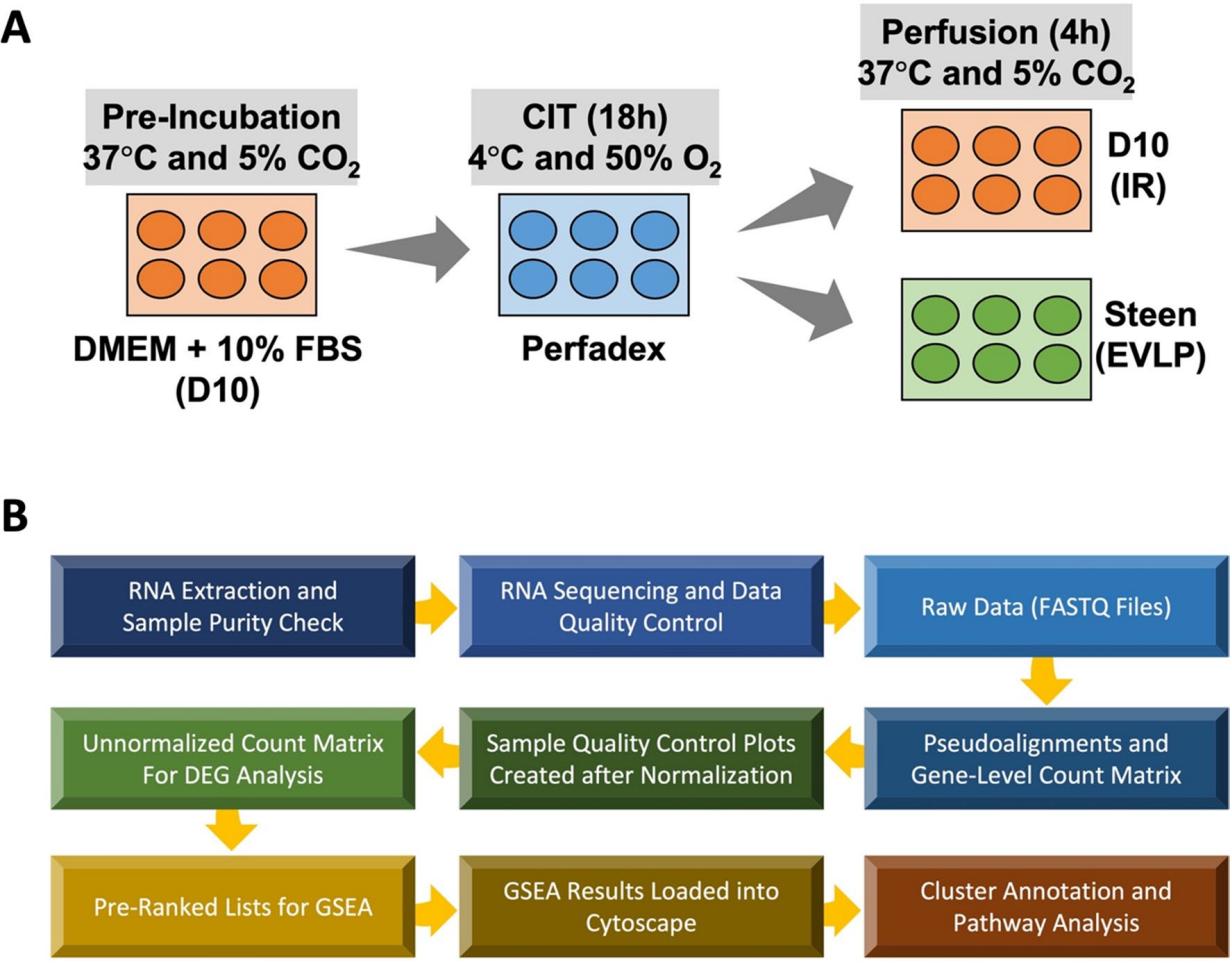
Experimental design and bioinformatics workflow. **A**. Human pulmonary microvascular endothelial cells (HPMEC) and human lung epithelial cells (BEAS-2B) were incubated with DMEM + 10% FBS (D10) at 37 °C until sub-confluent, and then preserved with lung preservation Perfadex^®^ solution at 4 °C with 50% O_2_ for 18 h cold ischemic time (CIT) to simulate static cold storage of donor lung. Cells were then switched to either D10 to simulate reperfusion during lung transplantation, or with Steen solution to simulate ex vivo lung perfusion (EVLP), respectively, for 4 h at 37 °C. **B**. Total RNA was extracted after CIT 18 h, reperfusion 4 h, or EVLP 4 h. After purity check, RNA samples were sequenced, and raw data (FASTQ files) were processed as indicated in the flow chart for differentially expressed gene (DEG) analysis and gene set enrichment analysis (GSEA)

The IR and EVLP cell culture models have been described in detail [16, 24]. Briefly, 30,000 cells/well were cultured in 6-well plates until sub-confluent in serum-containing DMEM + 10% FBS (D10) at 37 °C with 5% CO_2_, and then cold preservation was applied by replacing D10 with 4 °C Perfadex^®^ solution (Vitrolife; Englewood, CO) with 0.3 ml/L Tham and 0.6 ml/L CaCl_2_, and cells were stored in a sealed chamber filled with 50% O_2_ for 18 h to mimic cold ischemic time (CIT). Lastly, IR model had Perfadex^®^ solution replaced with D10, while EVLP model used Steen solution (XVIVO; Göteborg, Sweden), both perfused at 37 °C with 5% CO_2_ for 4 h (Fig. 1A).

### RNA samples preparation and bulk RNA-sequencing

There were 3 groups of conditions (i.e., CIT 18 h, D10 4 h, and Steen 4 h) for each cell type, and each group had 4 technical repeats, thus total 24 samples were analyzed. All wells were washed twice with PBS. Then 1 mL trypsin was added to each well for 2 min at 37 °C to detach the cells, and 1 mL D10 medium was added for each well to neutralize trypsin activity. After centrifugation at 250 g for 5 min, pellets were washed with PBS twice with centrifugation, the final pellets were snap-frozen at − 80 °C before usage.

RNeasy Mini Kit was used for RNA isolation according to the manufacturer recommendation (Cat. No.74904, QIAGEN; Hilden, Germany). The isolated RNA samples were purified with PureLink^™^ DNase Kit (Cat. No. 12185-010, Invitrogen; Carlsbad, CA). RNA purity was measured using the ratio of absorbance at 260 nm/280 nm for quality control. The RNA samples were sent to Princess Margaret Genomics Centre (Toronto, Canada) for bulk RNA-sequencing. 200 ng of total RNA per sample was used for library preparation, according to Illumina Truseq Stranded total RNA (Ribo Zero Gold TruSeq Stranded Total RNA Reference Guide available online: https://sapac.support.illumina.com/downloads/truseq-stranded-total-rna-reference-guide-1000000040499.html). Samples were sequenced on an Illumina NovaSeq 6000 (San Diego, CA) after assessment of samples using BioAnalyzer (Agilent; Santa Clara, CA, USA) for RNA integrity number (RIN), TapeStation (Agilent) for RNA library size confirmation and checking for adapter dimers, and qPCR for sequencing adjusting based on final concentrations of the samples. Each sample was sequenced targeting 40 million reads, with all sequences of 101-base-pair in length. Reads were aligned to the reference human transcriptome “hsapiens_gene_ensembl” (version 107) using kallisto v0.48.0.

### Differential gene expression analysis and pathway analysis

Kallisto was used to quantify transcript abundance estimates, which were then summarized at the gene level using tximport to generate count tables [26]. Differentially expressed genes (DEG) were computed using the DESeq2 R package, which utilizes an internal normalization system of the median of ratios method [27]. Any gene that had a sum expression count lower than 10 across 24 samples was excluded for further analysis. Principal component analysis (PCA) plots and a sample-to-sample heatmap were generated to visualize the distribution or clustering of the samples. A statistically significant DEG was defined by having a false discovery rate (FDR) adjusted p-value below 0.05 and an absolute log2 fold change value larger than 0.5.

Genes were pre-ranked based on their p-values and the sign of log2 fold change values to generate a list. Then we used gene set enrichment analysis (GSEA) software to generate lists of gene sets with their corresponding enrichment scores and FDR-adjusted p-values [28]. Gene sets with FDR-adjusted p-values below 0.05 were visualized and clustered with EnrichmentMap and AutoAnnotate in Cytoscape software [29–31]. Subsequently, pathway clusters with less than four nodes (i.e., gene sets) were filtered out (Fig. 1B).

## Results

Endothelial and epithelial cells are major parenchymal cells in the donor lung. The stress induced by cold preservation and warm reperfusion is mainly applied to these cells. Saren et al. studied the responses of HPMEC and BEAS-2B cells to simulated IR with microarray and find reperfusion 4 h after 18 h CIT induced significant changes of transcriptomic profiles in both cell types [16]. Therefore, in the present study, we also used 18 h of CIT to ensure sufficient damage to the cells and consider as the baseline for comparisons against 4 h perfusion samples within each cell line. We chose 4 h as the endpoint of warm reperfusion or EVLP to reflect the animal models of IR injury in LTx and current clinical EVLP practice that usually lasts 4–6 h [5, 32, 33].

### IR and EVLP induced differential changes in gene expressions

**Fig. 2:**
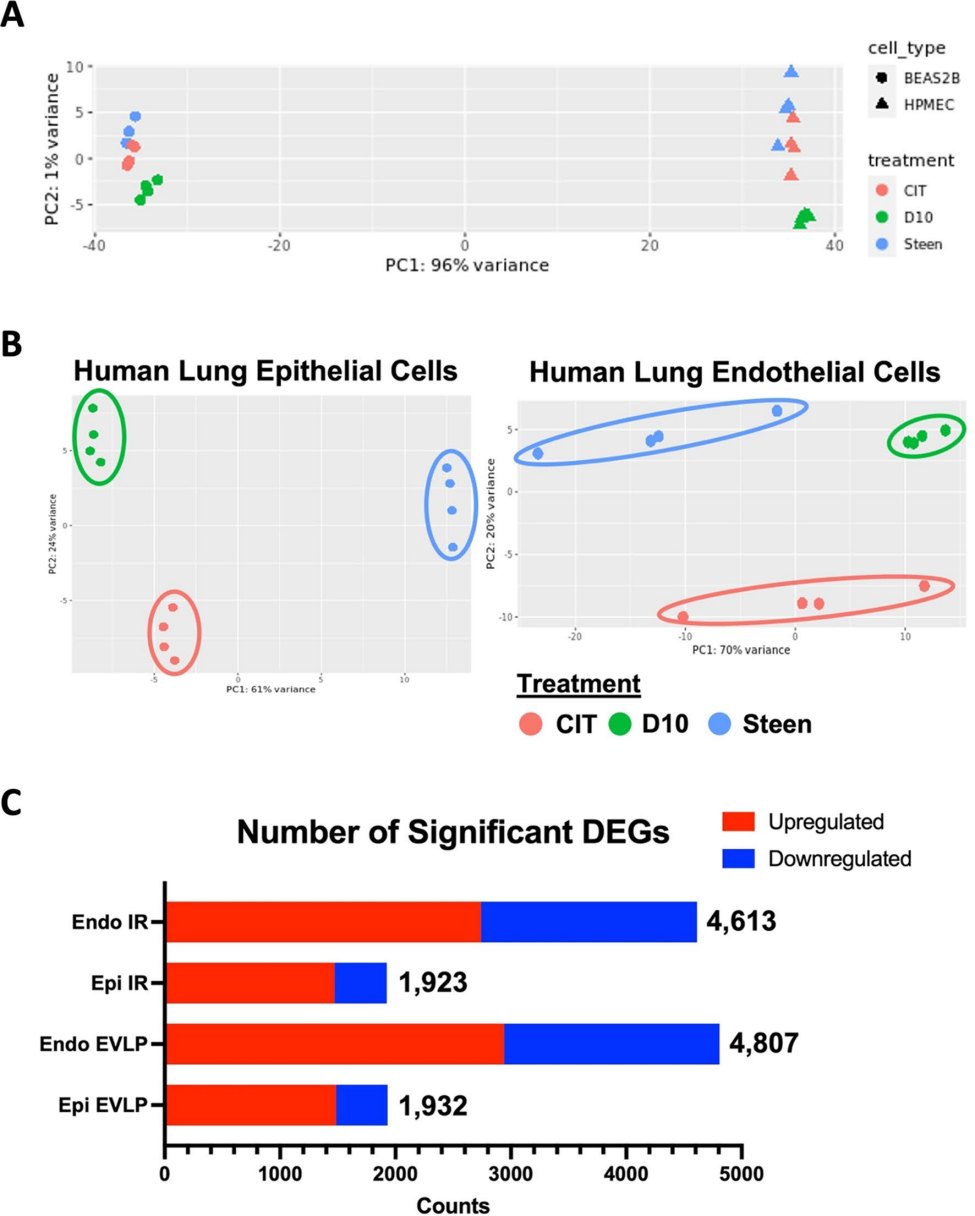
Principal component analysis (PCA) plots and a bar graph of significant differentially expressed genes (DEGs) in human lung endothelial and epithelial cells after simulated ischemia–reperfusion (IR) or ex vivo lung perfusion (EVLP). **A**. PCA plot shows separation between epithelial and endothelial cell samples. **B**. PCA plots show three distinct clusters of CIT (red), D10 (green), and Steen (blue) samples. **C**. Compared with cells undergone CIT 18 h, endothelial cells had higher numbers of significant DEGs compared to those of epithelial cells in both IR and EVLP models. Statistical cut-off for significant DEGs: FDR-adjusted p-value < 0.05 and |log2 fold change|> 0.5

The PCA plot of all 24 samples showed that the epithelial cell samples were clustered on one side and the endothelial cell samples on the opposite end (Fig. 2A), and a similar observation was found by Saren and colleagues with microarray [16]. In addition, clusters of CIT, D10 (i.e., IR model), and Steen samples (i.e., EVLP model) showed distinct separation (Fig. 2B**)**. Additional file 1: Figure S1 contains a heatmap and a boxplot showing overall distribution among the samples.

After pre-filtering, there were 21,358 genes that were annotated. DESeq2 analyses showed that the numbers of statistically significant DEGs (FDR-adjusted p-values < 0.05 and |log2 fold change|> 0.5) were higher in endothelial IR (n = 4613) and EVLP models (n = 4807) compared to those in epithelial cells IR (n = 1923) and EVLP samples (n = 1932), as shown in Fig. 2C. Additional file 1: Figures S2, S3 show FDR-adjusted p-value histograms and volcano plots of the four models.

### IR activated pro-inflammatory signaling and vascular process in endothelial cells

Using GSEA, 835 significant gene sets were found in endothelial IR model (Additional file 1: Table S1). The ranked lists of upregulated (positive enrichment scores) and downregulated (negative enrichment scores) gene sets were clustered in Cytoscape using the EnrichmentMap pipeline (https://enrichmentmap.readthedocs.io/en/latest/).

**Fig. 3:**
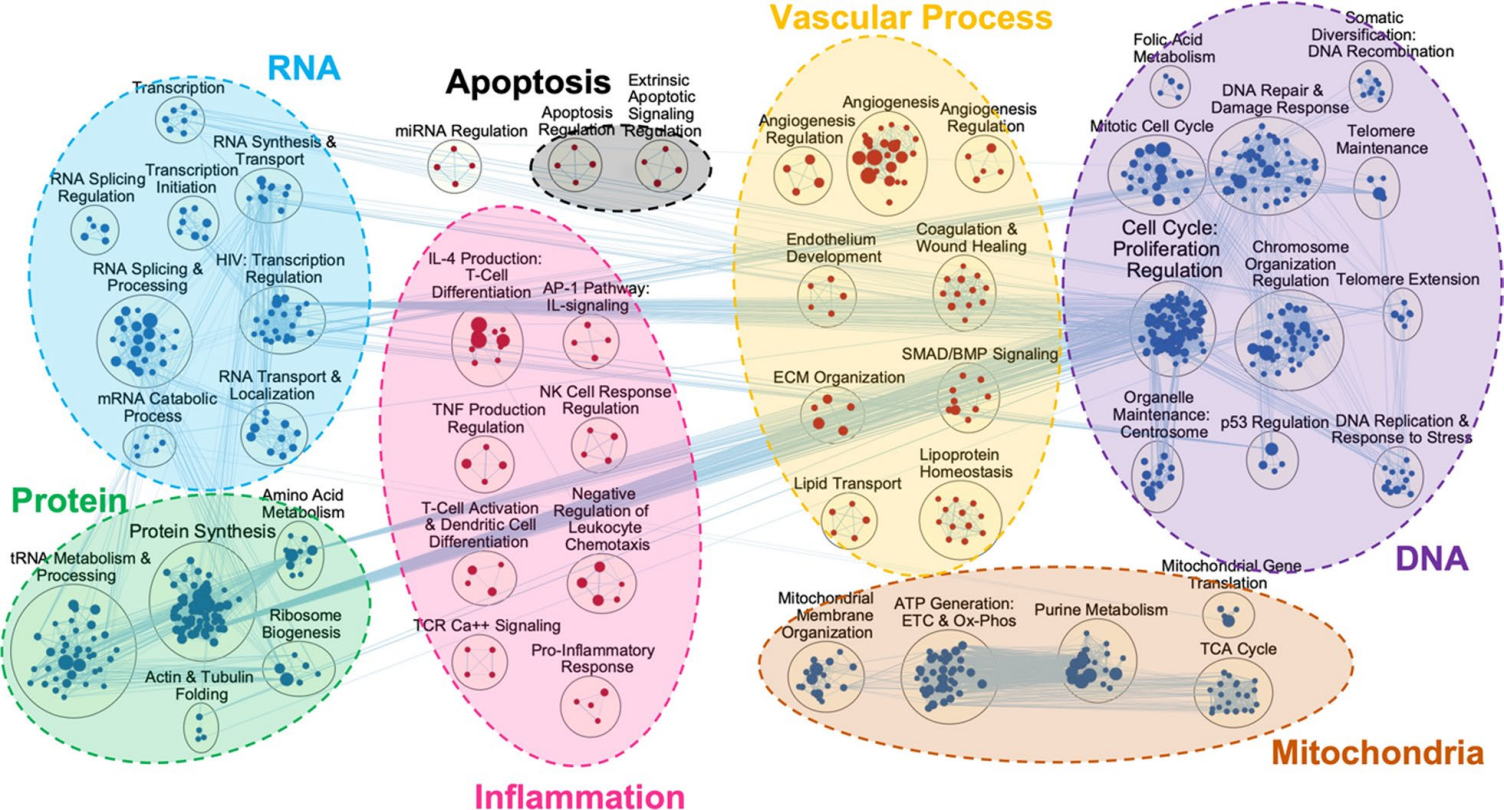
Simulated ischemia–reperfusion (IR) in lung transplantation induces inflammation and cell type specific gene expression in human lung endothelial cells. Network visualization of significant pathways are shown using EnrichmentMap and AutoAnnotate in Cytoscape software. In human pulmonary microvascular endothelial cells (HPMEC), IR induced increase in inflammation, vascular process, and apoptosis themes of pathway clusters (red nodes). On the other hand, clusters in DNA, RNA, protein, and mitochondria themes were downregulated (blue nodes)

In endothelial cells, IR model exhibited three main themes of upregulated clusters: inflammation, vascular process, and apoptosis (Fig. 3**)**. Inflammation theme included interleukin (IL) 4 production, T-cell activation and dendritic cell differentiation, and tumor necrosis factor (TNF) production regulation, while vascular process included angiogenesis regulation, endothelium development, coagulation and wound healing, as well as extracellular matrix organization. Four main themes of downregulated clusters were DNA, RNA, protein, and mitochondria. In mitochondria theme, there were ATP generation, tricarboxylic acid cycle, and purine metabolism clusters, while the DNA theme involved cell cycle, chromosome organization regulation, telomere extension, and DNA replication and response to stress clusters. RNA theme included transcription, RNA splicing regulation, transcription initiation, RNA synthesis and transport, and mRNA catabolic process clusters. Lastly, protein theme involved amino acid metabolism, protein synthesis, tRNA metabolism and processing, actin and tubulin folding, and ribosome biogenesis clusters. Overall, IR activated the endothelial cells to increase pro-inflammatory signaling, apoptosis, and vascular processes, while decreasing genes in metabolism pathways.

### IR increased inflammatory signaling and epithelial process in epithelial cells

**Fig. 4:**
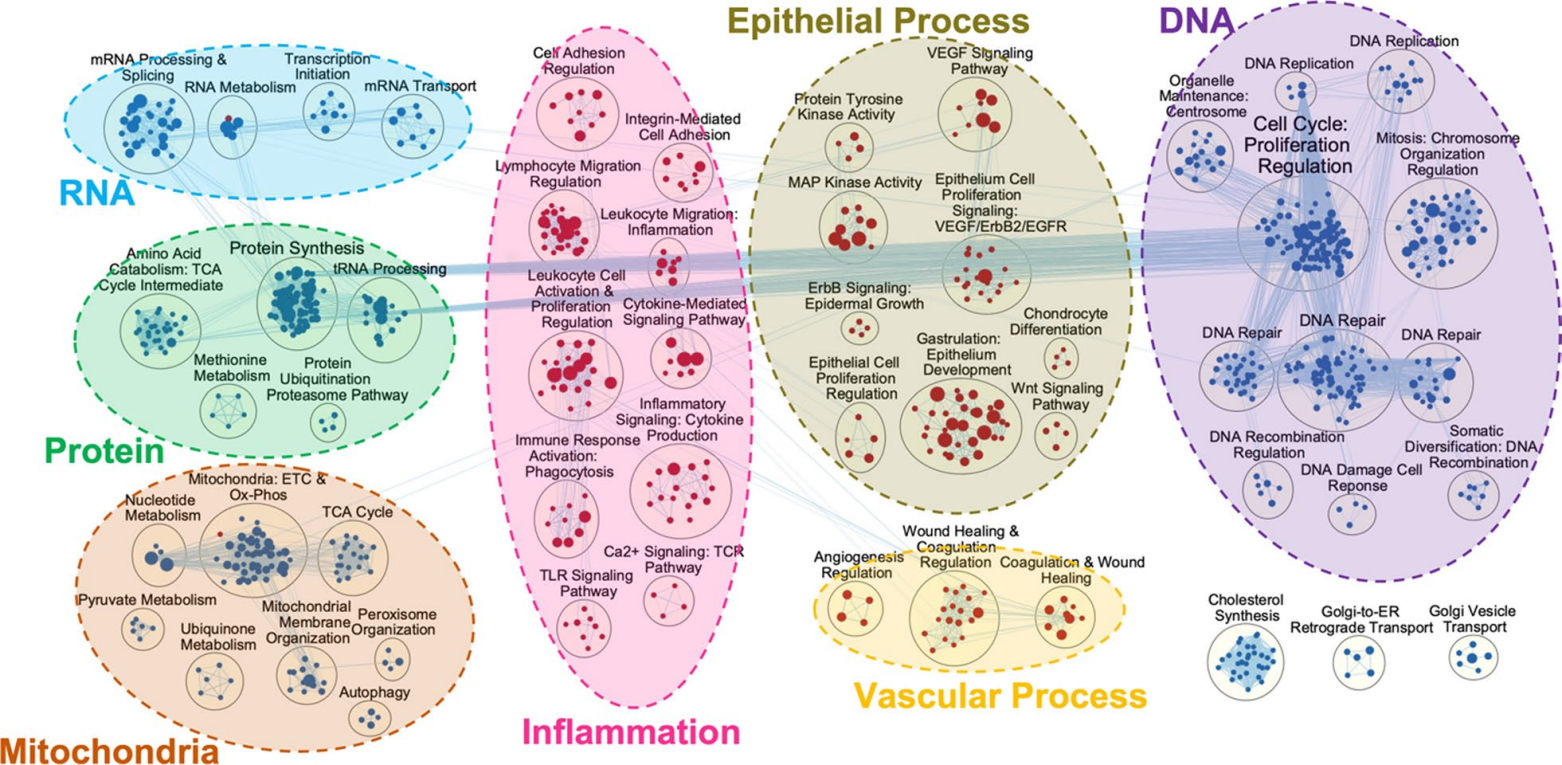
Simulated ischemia–reperfusion (IR) in lung transplantation induces inflammation and cell type specific gene expression in human lung epithelial cells. Network visualization of significant pathways are shown using EnrichmentMap and AutoAnnotate in Cytoscape software. Human epithelial cells (BEAS-2B) IR model showed upregulation (red nodes) in inflammation, vascular process, and epithelial process themes, and downregulation (blue nodes) in DNA, RNA, protein, and mitochondria themes

In epithelial IR model, there were 1040 significant gene sets (Additional file 1: Table S1). IR upregulated pathways involved in inflammation, epithelial process, and vascular process, whereas DNA, RNA, protein, and mitochondria themes were downregulated (Fig. 4), similar to that seen in endothelial IR model. Inflammation theme included cytokine-mediated signaling, toll-like receptor (TLR) signaling, T-cell receptor (TCR) signaling, lymphocyte migration, leukocyte cell activation, and cell adhesion regulation. In epithelial process theme, there were epithelial cell proliferation regulation, epithelium development, and epidermal growth clusters, while vascular process theme composed of angiogenesis, wound healing and coagulation clusters. Downregulated clusters in DNA, RNA, and protein themes were comparable to those observed in endothelial IR model, where the pathways involved the regulation of each biomolecule’s production, repair, metabolism, and/or transport. Mitochondria theme also similarly reflected downregulation of ATP generation.

### EVLP did not activate inflammatory signaling in endothelial and epithelial cells

Interestingly, in both endothelial and epithelial cells, 4 h perfusion with acellular Steen solution did not induce upregulation of any significant pathways. There was only one significantly upregulated gene set in endothelial EVLP model from GSEA result, and it was annotated as “anterior/posterior pattern specification” (Additional file 1: Table S1). When loaded into Cytoscape, however, this gene set did not form a significant cluster with other pathways. Thus, only downregulated gene clusters were visualized.

**Fig. 5:**
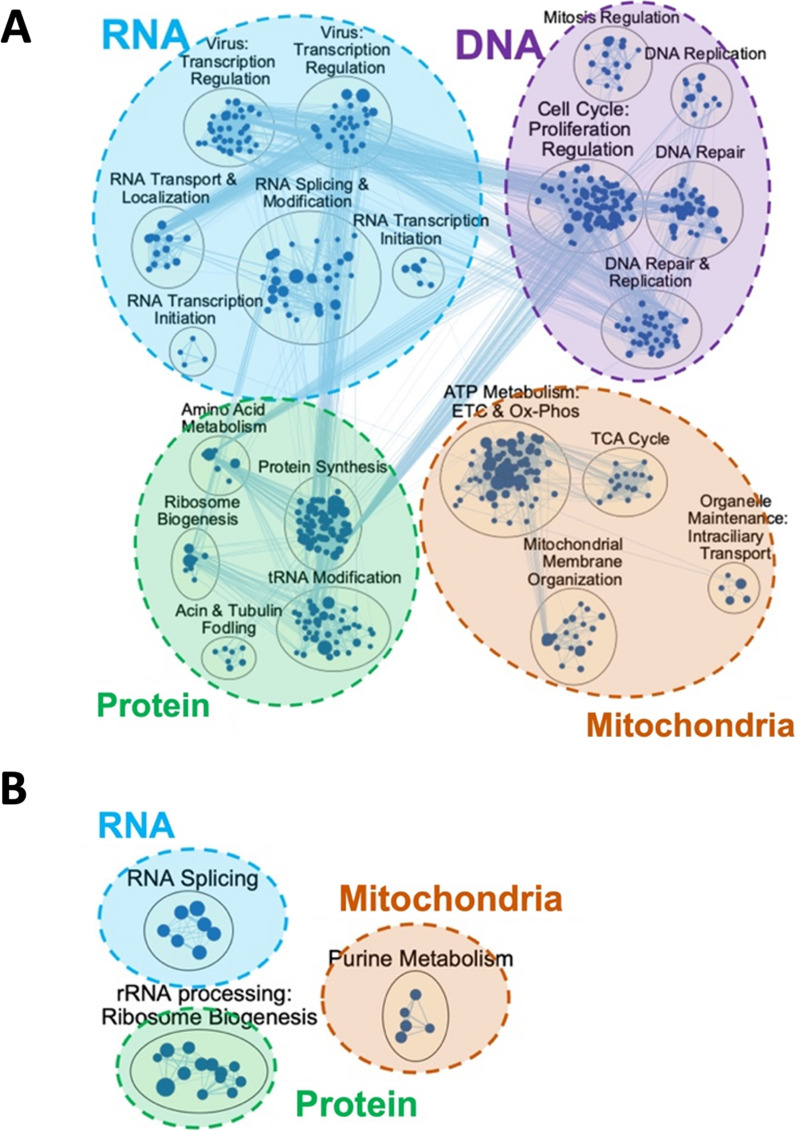
Simulated ex vivo lung perfusion (EVLP) did not induce genes related to inflammation and cell death in human lung endothelial and epithelial cells. **A**. In human pulmonary microvascular endothelial cells (HPMEC), simulation of EVLP led to downregulation of gene clusters in DNA, RNA, protein, and mitochondria themes, and no upregulated pathways were observed. **B**. In human lung epithelial cells (BEAS-2B), EVLP model showed downregulation in three pathways that belonged to RNA, protein, and mitochondria themes. Again, there were no upregulated pathways

In endothelial cells, downregulated themes of DNA, RNA, protein, and mitochondria were observed. DNA theme included mitosis regulation, DNA replication, cell cycle, and DNA repair clusters. RNA theme contained transcription regulation, RNA splicing & modification, and RNA transport and localization clusters. Protein theme had amino acid metabolism, protein synthesis, ribosome biogenesis, organelle intraciliary transport, actin and tubulin folding, and tRNA modification clusters. Lastly, mitochondria theme contained ATP metabolism, TCA cycle, and mitochondrial membrane organization (Fig. 5A**)**. Epithelial cells exhibited only three clusters, where RNA splicing cluster belonged to RNA theme, purine metabolism to mitochondria theme, and ribosome biogenesis to protein theme (Fig. 5B). We witnessed more diverse downregulated signaling involved in cell metabolism in the endothelial cells than in the epithelial cells after CIT 18 h and 4 h of warm perfusion with acellular Steen solution.

## Discussion

Similar to what we have observed in human lung transplants studies, we found that IR simulation activated pathways related to inflammatory responses in human lung endothelial and epithelial cells. On the other hand, different from what we have seen in human lungs, our EVLP models did not involve inflammatory responses. The cell culture models will be helpful for interpreting the direct and indirect responses of these lung parenchymal cell types to IR and EVLP conditions in the human lung grafts.

### IR condition activates inflammatory responses directly at cellular levels

Wong et al. analyzed the transcriptomic changes in human lung biopsies before and after transplantation, and found upregulated themes of inflammation, cell death, and heat stress and downregulated themes of metabolism and protein synthesis [12]. The lung allografts contain a wide range of cell types, such as donor endothelial and epithelial cells, and macrophages, monocytes, neutrophils, and natural killer cells from both donor and recipient. Various interactions among these cells have been reported to contribute to IR injury [34]. The contribution of lung endothelial and epithelial cells to inflammatory responses cannot be determined with the bulk RNA studies in lung tissues.

**Fig. 6:**
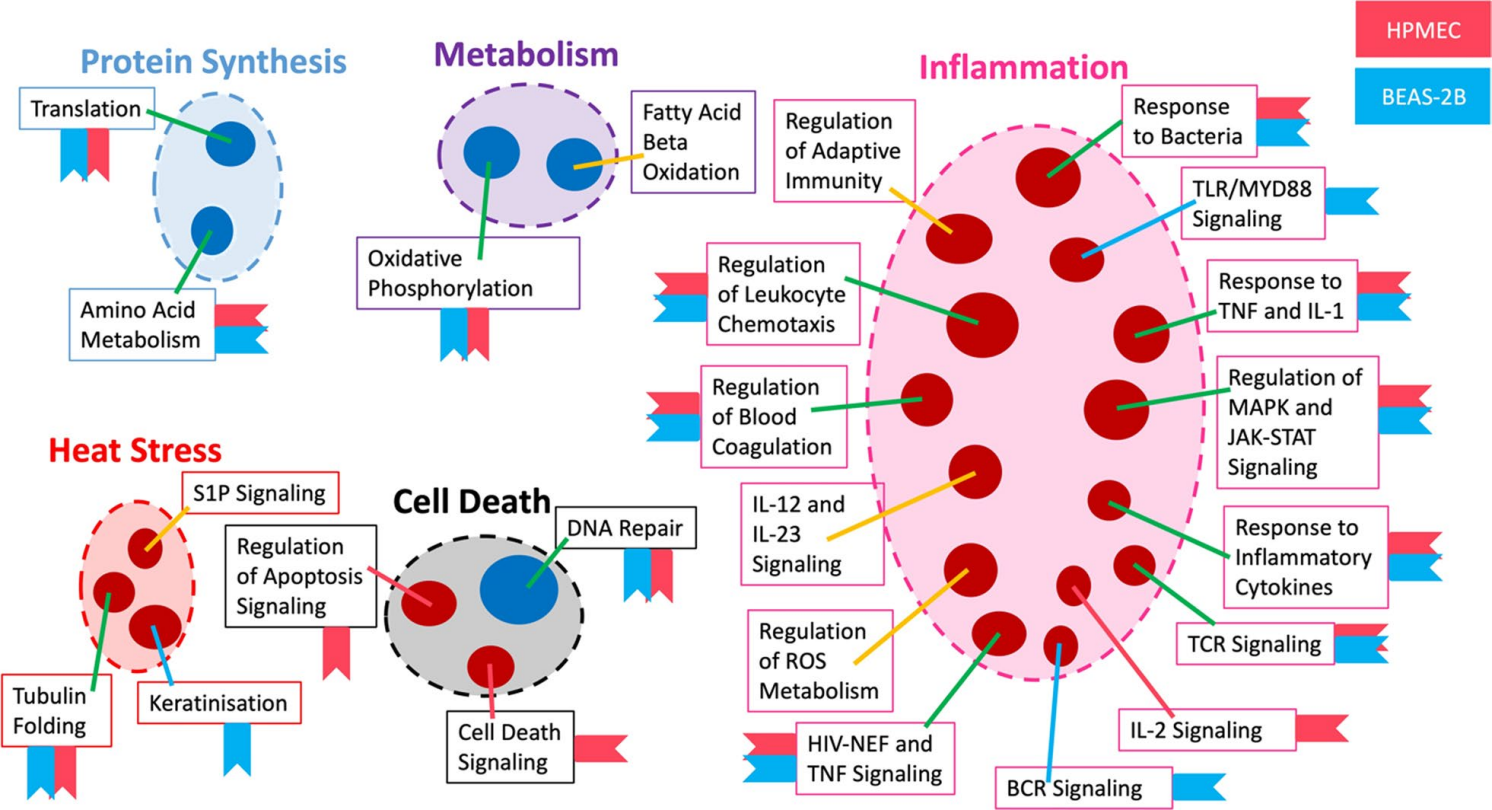
Simulated ischemia–reperfusion (IR) in human lung endothelial and epithelial cell cultures induced similar pathways as reported in human lung transplants (11). Dark red nodes indicate upregulated pathway clusters, while blue nodes indicate downregulated clusters observed in human lung transplants. Blue-labeled tags are attached to the pathway boxes if identified in epithelial cells (BEAS-2B) and pink-labeled tags if identified in endothelial cells (HPMEC)

In both lung endothelial and epithelial IR models, we identified upregulation of inflammatory signals that were seen in human lung transplant samples, such as response to bacteria, response to TNF and IL-1, regulation of mitogen-activated protein kinase (MAPK) and Janus kinase (JAK)-signal transducer and activator of transcription protein (STAT) signaling, response to inflammatory cytokines, TCR signaling, human immunodeficiency virus (HIV)-negative regulatory factor (NEF) and TNF signaling, regulation of blood coagulation, and regulation of leukocyte chemotaxis. On the other hand, regulation of apoptosis signaling, cell death signaling, and IL-2 signaling were only seen in endothelial cells, whereas response to TLR/myeloid differentiation primary response 88 (MyD88) signaling and B-cell receptor (BCR) signaling were only seen in epithelial cells. Similarly, downregulation of pathways in amino acid metabolism, protein translation, oxidative phosphorylation, and DNA repair, seen in human lung transplants, were also detected in both human lung endothelial and epithelial cells (Fig. 6). These cell culture results indicate that human lung endothelial and epithelial cells may play an essential role in IR-induced inflammatory responses. Activation of inflammatory responses in these residential cells may prime the donor lung to further interact with donor and recipient immune cells for subsequent tissue injury. The pathways that were not detected or found to be significant in our cell culture models, such as regulation of reactive oxygen species (ROS) metabolism, IL-12 and IL-23 signaling, regulation of adaptive immunity, Sphingosine-1-phosphate (S1P) signaling and fatty acid beta-oxidation (Fig. 6) could be attributed to other cell types and/or to the interactions among different cell types.

### Acellular EVLP perfusate does not activate inflammatory responses in cell culture models

On the contrary, after cold preservation, the simulated EVLP did not show enriched pathways related to pro-inflammatory responses in both cell types, which was the opposite to what Wong et al. observed in the human lung biopsies after EVLP [12]. In animal models and clinical studies, inflammatory mediators are released from the donor lungs into the EVLP perfusate, and using cytokine filters to reduce the levels of inflammatory mediators ameliorated inflammation and improved lung graft function during EVLP and post-LTx [35–38]. In addition, Yeung et al. examined gene expression profiles of human donor lungs during EVLP, and found that EVLP may improve donor lung function through the washout of leukocytes and facilitate innate mechanisms of repair [39]. In our EVLP cell culture model, these inflammatory mediators and other types of cells are not included in the system, thus this may partially explain the lack of pathways in inflammation and cell death.

However, in our IR models, the inflammatory mediators and other cell types are also missing, but the serum and nutrients in the medium may be activating genes related to endothelial and epithelial cell functions and those involved in the pro-inflammatory responses. It has been shown that simulated reperfusion condition in cell culture can increase the release of pro-inflammatory cytokines and induce apoptosis and necroptosis, and these mechanisms identified in the cell culture models have been validated in animal models or human lung samples [17–19, 22, 23, 32]. The Steen solution, which only contains buffered electrolytes, glucose, albumin and dextran 40, may not fully activate genes related to endothelial/epithelial functions and pathways related to inflammation and cell death. The acellular Steen solution used for EVLP may provide a protective milieu for donor lung cells to recover from the stress induced by the hypothermic and ischemic condition. These results support a recent literature review on EVLP clinical trials reporting a reduced incidence of PGD following LTx for donor lungs assessed with EVLP [9].

### Differential responses of endothelial and epithelial cells to IR and EVLP

In both IR and EVLP models, human pulmonary microvascular endothelial cells had nearly double the number of DEGs than that of human lung epithelial cells. Moreover, between the EVLP models, the number of gene sets downregulated in the endothelial cells was far more than that of the epithelial cells. These results suggest that endothelial cells might be more responsive to cold preservation and warm (re)perfusion conditions. On the contrary, Saren et al.’s study that directly compared between the two cell types undergoing cold ischemia and warm reperfusion has reported significantly different transcriptomic profiles for each cell type, and the epithelial cells were suggested to be more sensitive to IR conditions than endothelial cells, due to the disappearance of epithelial-specific clusters after IR [16]. However, the disappearance of epithelial-specific pathways may be due to the disorganized regulation of genes that did not reach statistical significance in pathway analyses, whereas the number of significant DEGs may reflect a better overview of the responsiveness of each cell type. Even though both endothelial cells and epithelial cells are abundant in the lung, their responses to cold donor lung preservation and warm reperfusion or EVLP can be distinctive. Therefore, cell-type-specific biomarkers and therapeutic targets should be explored in the future studies.

### Limitations and future directions

Interestingly, a vascular process theme was observed in our epithelial IR model. Because of its bronchial epithelial origin, BEAS-2B cells have been widely used in lung cell studies. However, very few studies have reported their non-epithelial features, some of which are comparable with those seen in human mesenchymal stem cells [40]. Whether the gene set regulations in the vascular process theme of epithelial IR model can be found in vivo needs to be determined.

To compare with transcriptomic data from human lung transplant, we used GSEA and Cytoscape for pathway analyses. Ingenuity pathway analysis (IPA) is another commonly used method for transcriptomics studies. The bulk RNA sequencing data collected in this study can be analyzed with various methods and should be explored further.

In the present study, we used fetal bovine serum in cell culture medium. Even though this is a common practice in cell biology studies, considering the transplant setting, using bovine serum for human cell culture, may have different stimulatory effects from human blood. This should be considered as a potential cofounding factor.

Moreover, due to the nature of mono cell cultures of two-dimensions, there were no interactions among different cell types, which would have induced more diverse signals after warm (re)perfusion. Hence, whether our results can be reproduced in three-dimension cell cultures or co-cultures should be explored further. In addition, both IR and EVLP models did not include flow and ventilation, which are important factors for lung cell physiology [41, 42]. Advancements in development of the experimental cell culture models representing in vivo lung environment may provide a closer reflection of molecular responses in clinical scenarios.

Lastly, because the findings are at a transcriptional level, we are yet to investigate whether proteomics and metabolomics data would support or rebut our current findings. In future studies, a more developed version of cell culture models simulating IR and EVLP and a multi-omics approach would yield a more solid proposition on the mechanisms underlying IR and acellular EVLP.

In conclusion, using bulk RNA sequencing technique, we profiled transcriptomic changes of human lung endothelial and epithelial cells in simulated IR and EVLP models. The similarity of inflammatory responses observed in these cell cultures and in human lung transplants suggests that lung endothelial and epithelial cells can play a huge role in IR-induced injury of the lung grafts. The absence of inflammatory responses in our EVLP models suggests that the lack of serum components in the acellular Steen solution may limit the activation of pro-inflammatory signals, thus supporting the protective role of clinical EVLP.

### Supplementary Information

**Additional file 1: Table S1.** Numbers of significant gene sets in the ischemia-reperfusion (IR) or ex vivo lung perfusion (EVLP) models of human lung endothelial (HPMEC) and epithelial (BEAS-2B) cells. **Figure S1.** Quality control plots. **A**. Boxplot of all 24 samples before normalization. **B**. A sample-to-sample heatmap of all samples after variance stabilizing transformation. **Figure S2.** Differentially expressed gene analysis results for human lung endothelial cells (HPMEC) IR model (D10 vs. CIT) and EVLP model (Steen vs. CIT). **Figure S3.** Differentially expressed gene analysis results for human lung epithelial cells (BEAS-2B) IR model (D10 vs. CIT) and EVLP model (Steen vs. CIT).

## Data Availability

The raw and processed data through DESeq2 is available at GEO: GSE228488 (https://www.ncbi.nlm.nih.gov/geo/query/acc.cgi?acc=GSM7122628).

## Abbreviations

BCR: B-cell receptor
CIT: Cold ischemic time
D10: DMEM + 10% FBS
DEG: Differentially expressed gene
EVLP: Ex vivo lung perfusion
GSEA: Gene set enrichment analysis
HIV: Human immunodeficiency virus
IL: Interleukin
IR: Ischemia–reperfusion
JAK: Janus kinase
LTx: Lung transplantation
MYD: Myeloid differentiation primary response
NEF: Negative regulatory factor
PCA: Principal component analysis
PGD: Primary graft dysfunction
ROS: Reactive oxygen species
S1P: Sphingosine-1-phosphate
STAT: Signal transducer and activator of transcription protein
TCA: Tricarboxylic acid
TCR: T-cell recepto
TLR: Toll-like receptor
TNF: Tumor necrosis factor
