# Supplementary material for: Acellular ex vivo lung perfusate silences pro-inflammatory signaling in human lung endothelial and epithelial cells
Source: J Transl Med. 2023 Oct 17;21:729. doi: 10.1186/s12967-023-04601-w (PMC10580637; doi:10.1186/s12967-023-04601-w)
Supplement: Supplementary file 1 — Additional file 1: Table S1. Numbers of significant gene sets in the ischemia-reperfusion (IR) or ex vivo lung perfusion (EVLP) models of human lung endothelial (HPMEC) and epithelial (BEAS-2B) cells. Figure S1. Quality control plots. A. Boxplot of all 24 samples before normalization. B. A sample-to-sample heatmap of all samples after variance stabilizing transformation. Figure S2. Differentially expressed gene analysis results for human lung endothelial cells (HPMEC) IR model (D10 vs. CIT) and EVLP model (Steen vs. CIT). Figure S3. Differentially expressed gene analysis results for human lung epithelial cells (BEAS-2B) IR model (D10 vs. CIT) and EVLP model (Steen vs. CIT). [file 12967_2023_4601_MOESM1_ESM.docx]

**Supplementary Materials**

**Table S1. Numbers of significant gene sets in the ischemia-reperfusion (IR) or ex vivo lung perfusion (EVLP) models of human lung endothelial (HPMEC) and epithelial (BEAS-2B) cells.**


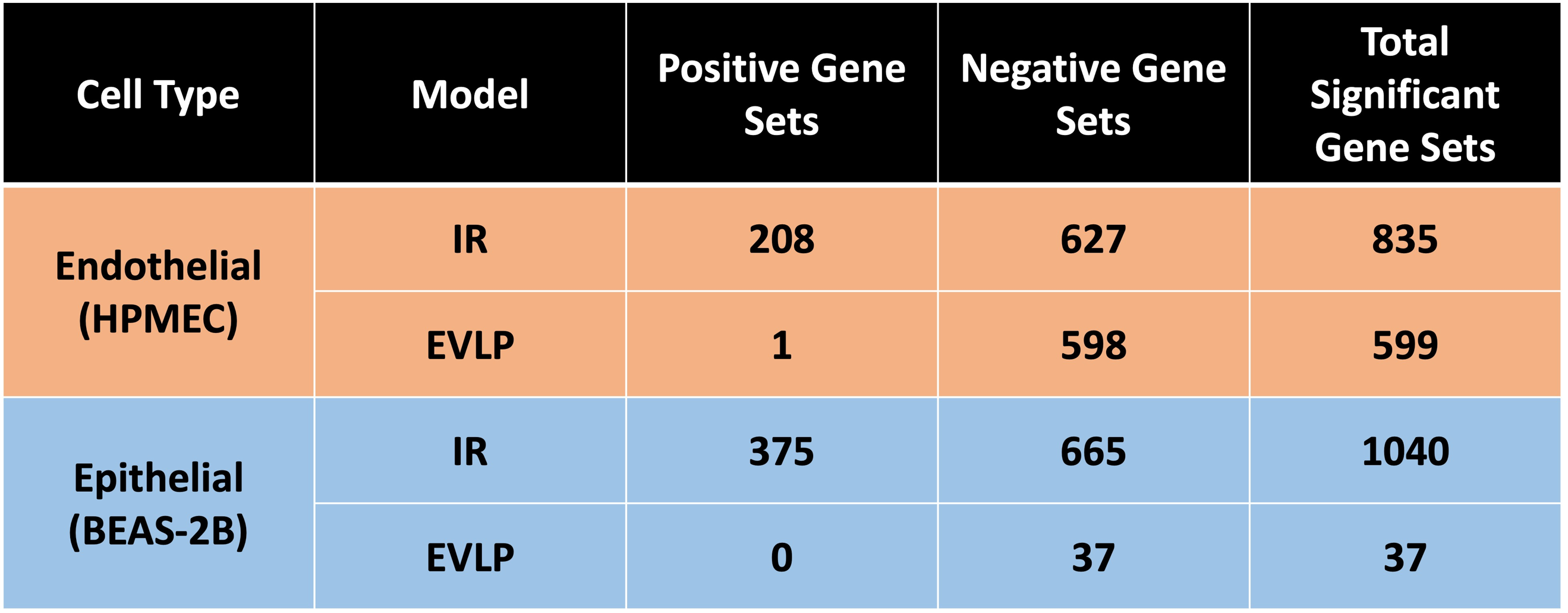


Among the ranked lists of gene sets returned by GSEA (gene set enrichment analysis), the numbers of significant gene sets were determined prior to loading the entire lists into Cytoscape’s EnrichmentMap application. IR models had higher total numbers of significant gene sets compared to EVLP models for both endothelial and epithelial cells. Overall, each model had more down-regulated gene sets than up-regulated ones. The cut-off for significant gene sets was FDR-adjusted p-value < 0.05.

**A**

**
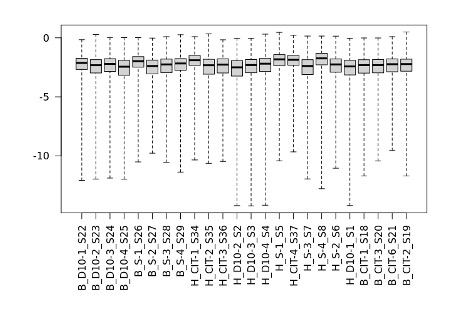
**

**B**

**
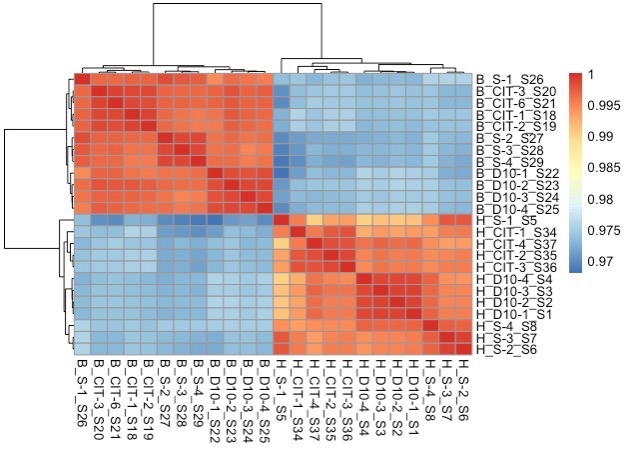
**

**Figure S1. Quality control plots. A.** Boxplot of all 24 samples before normalization. **B.** A sample-to-sample heatmap of all samples after variance stabilizing transformation.

**A**

**
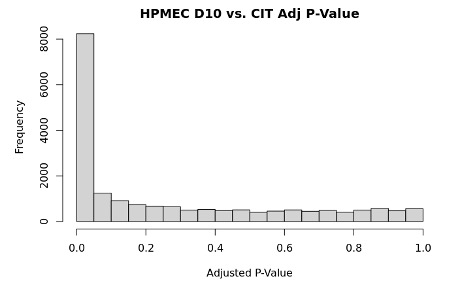

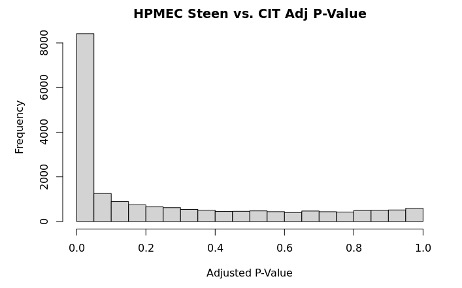
**

**B**

**
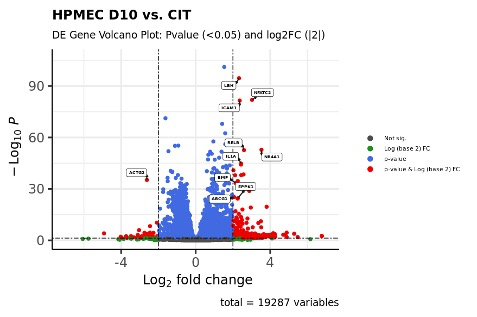

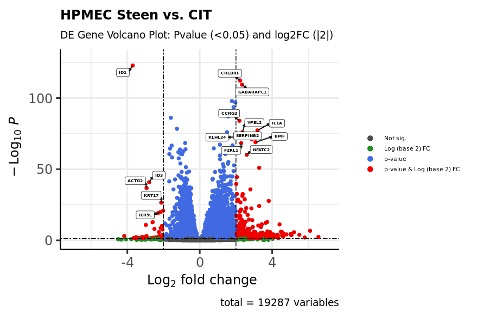
**

**Figure S2. Differentially expressed gene analysis results for human lung endothelial cells (HPMEC) IR model (D10 vs. CIT) and EVLP model (Steen vs. CIT). A.** Histogram of FDR-adjusted p-values for each model. **B.** Volcano plots exhibiting differentially expressed genes (DEGs) populations.

**A**

**
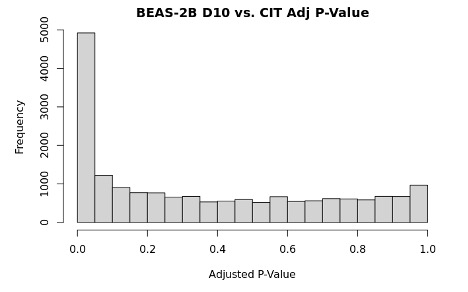

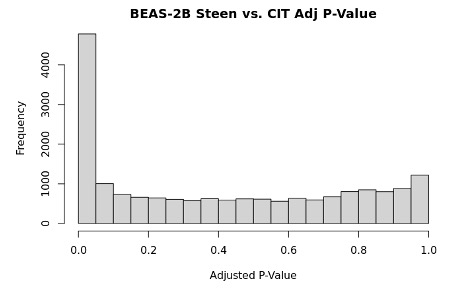
**

**B**

**
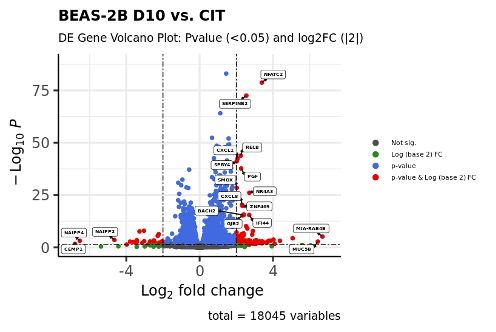

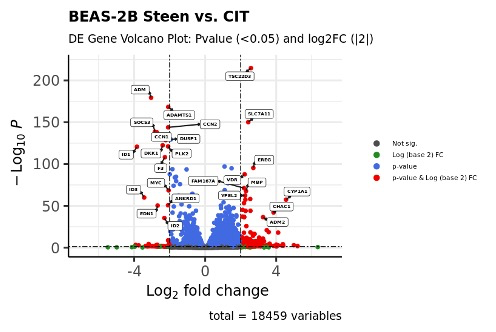
**

**Figure S3. Differentially expressed gene analysis results for human lung epithelial cells (BEAS-2B) IR model (D10 vs. CIT) and EVLP model (Steen vs. CIT). A.** Histogram of FDR-adjusted p-values for each model. **B.** Volcano plots exhibiting differentially expressed genes (DEGs) populations.
